# P001 Severe vitamin C deficiency presenting as a mimic of inflammatory myositis

**DOI:** 10.1093/rap/rkag113.031

**Published:** 2026-11-03

**Authors:** Shabnam Tariq, Sameena Khalid, Melisa Topchu

**Affiliations:** Sheffield Teaching Hospitals, Sheffield, United Kingdom; Department of Rheumatology, Sheffield Teaching Hospitals NHS Foundation Trust, Sheffield, United Kingdom; Rotherham General Hospital, Rotherham, United Kingdom

## Abstract

**Introduction:**

Severe micronutrient deficiency remains an important metabolic cause of musculoskeletal morbidity, but may not be considered early in rheumatology presentations. Vitamin C deficiency can cause spontaneous bleeding, soft tissue pain and functional decline. In this case, diffuse muscle oedema and perifascial signal abnormality on magnetic resonance imaging initially raised concern for inflammatory myositis. However, the overall clinical picture suggested a nutritional metabolic mimic rather than primary autoimmune muscle disease. This case is relevant because it highlights a reversible cause of significant musculoskeletal bleeding with misleading inflammatory-appearing imaging.

**Case description:**

We present the case of a wheelchair-dependent adult with chronic right-sided neurological weakness following resection of a fourth ventricle ependymoma, who presented with progressive atraumatic bruising and swelling of the right lower limb. The patient described enlargement of a posterior thigh haematoma without trauma, anticoagulant use, alcohol excess or known inherited bleeding disorder. Background history included iron deficiency anaemia and folate deficiency.

On examination, there was marked ecchymosis and swelling over the posterior right thigh extending towards the proximal calf. Knee flexion was restricted by pain and swelling, although extension and straight-leg raise were preserved. There was no compartment syndrome or clinically significant synovitis.

Blood tests showed severe anaemia, with haemoglobin 64 g/L compared with a previous baseline of 131 g/L. Erythrocyte sedimentation rate was 120 mm/hour and C-reactive protein was 69 mg/L. Creatine kinase was normal at approximately 35 U/L. Further investigations showed low iron, low folate, vitamin D less than 15 nmol/L and profound vitamin C deficiency, with serum concentration less than 3 micromol/L. Coagulation studies were normal. Autoimmune screening was negative, including antinuclear antibody, extractable nuclear antigen antibodies, anti-double stranded DNA antibody and myositis-specific antibodies. Complement levels were normal.

Computed tomography did not identify malignancy or an alternative bleeding source. Computed tomography angiography showed no active arterial bleeding or vascular abnormality. Magnetic resonance imaging demonstrated diffuse bilateral muscle oedema involving the posterior compartment musculature, perifascial signal abnormality, bilateral knee effusions and haemosiderin deposition consistent with resolving haemarthroses. Electromyography showed patchy fibrillation potentials in the gastrocnemius with low-amplitude polyphasic motor unit potentials, without evidence of a widespread peripheral neuropathy.

The patient received high-dose oral vitamin C, iron, folate and vitamin D replacement, and packed red blood cell transfusion. Bruising and swelling improved, inflammatory markers fell, and repeat imaging showed interval improvement.

**Discussion:**

This case fits within the metabolic bone disorder category as a nutritional metabolic presentation causing significant musculoskeletal morbidity and diagnostic uncertainty. The main challenge was that the magnetic resonance imaging appearances were suggestive of inflammatory myositis, but the clinical and biochemical picture did not support this diagnosis.

The presentation was dominated by spontaneous bleeding, with a large intramuscular haematoma, bilateral haemarthroses and severe anaemia. There was no clear history of progressive symmetrical proximal muscle weakness. Creatine kinase remained normal, autoimmune serology was negative and coagulation studies were normal. Although inflammatory myopathy cannot be excluded by a normal creatine kinase alone, the overall pattern made primary autoimmune muscle disease less likely.

Profound vitamin C deficiency provided a more coherent explanation. Vitamin C deficiency impairs collagen synthesis and causes capillary fragility, leading to bruising, soft tissue bleeding and intra-articular bleeding, often with normal clotting studies. The normal coagulation screen was therefore important, as the bleeding tendency in vitamin C deficiency reflects vascular fragility rather than a primary clotting abnormality. In this patient, chronic neurological disability and wheelchair dependence were likely relevant risk factors for poor nutritional intake. The coexisting iron, folate and vitamin D deficiencies also supported a broader nutritional problem.

The key clinical decision was to avoid treating the scan in isolation from the patient. Recognising the nutritional metabolic mimic avoided unnecessary immunosuppression and allowed targeted replacement therapy. The patient’s clinical improvement and interval improvement on repeat imaging supported haemorrhagic and reactive muscle change rather than primary inflammatory myositis.

This case prompts discussion around when nutritional screening should be included in the work-up of atypical suspected myositis, particularly where musculoskeletal bleeding, normal muscle enzymes and negative autoimmune serology occur together.

**Key learning points:**

• This case demonstrates that nutritional metabolic disease can present with significant musculoskeletal manifestations and may mimic inflammatory rheumatic disease. Muscle oedema and perifascial signal abnormality on magnetic resonance imaging are not specific for inflammatory myositis and should be interpreted alongside the clinical pattern, muscle enzymes, serology and the dominant presenting problem.

• Vitamin C deficiency remains relevant in current clinical practice. It should be considered in patients with unexplained bruising, intramuscular bleeding, haemarthrosis, poor wound healing or atypical musculoskeletal presentations, especially where there are risk factors for restricted intake, functional dependence or coexisting micronutrient deficiencies. Normal clotting studies should not falsely reassure clinicians where the clinical picture suggests vascular fragility or nutritional deficiency as a cause of bleeding.

• This case also highlights the risk of diagnostic anchoring. The imaging findings were impressive, but the normal creatine kinase, negative autoimmune screen, normal coagulation studies and spontaneous haemorrhagic presentation made inflammatory myositis less likely. Considering a nutritional mimic helped avoid inappropriate immunosuppression and unnecessary invasive investigation.

• The learning objective for the conference would be to discuss how rheumatologists approach suspected inflammatory myopathy when the imaging, clinical picture and blood tests do not align, and how early multidisciplinary input may help avoid unnecessary immunosuppression or invasive investigation. I would also be interested to explore whether nutritional screening should be considered earlier in patients with unexplained musculoskeletal bleeding, particularly in those with disability, dependency or evidence of wider micronutrient deficiency.

